# Retraction Note: Single-wall carbon nanohorns inhibited activation of microglia induced by lipopolysaccharide through blocking of Sirt3

**DOI:** 10.1186/s11671-023-03908-3

**Published:** 2023-10-17

**Authors:** Lihong Li, Jinqian Zhang, Yang Yang, Qiang Wang, Li Gao, Yanlong Yang, Tao Chang, Xingye Zhang, Guoan Xiang, Yongmei Cao, Zujin Shi, Ming Zhao, Guodong Gao

**Affiliations:** 1 Department of Neurosurgery, Tangdu Hospital, Fourth Military Medical University, Xi’an, 710038 China; 2 Institute of Infectious Diseases, Beijing Ditan Hospital, Capital Medical University, Beijing, 100015 China; 3 Department of General Surgery, The Second People’s Hospital of Guangdong Province, Guangzhou, 510515 China; 4 International Mongolian Medical Hospital of Inner Mongolia, Hohhot, 010065 China; 5 Beijing National Laboratory for Molecular Sciences, State Key Laboratory of Rare Earth Materials Chemistry and Applications, College of Chemistry and Molecular Engineering, Peking University, Beijing, 100871 China; 6 Department of Chemical Biology, School of Pharmaceutical Sciences, Peking University, Beijing, 100191 China

**Retraction Note: Nanoscale Research Letters 2013, 8:100** 10.1186/1556-276X-8-100

The Editors-in-Chief have retracted this article. Concerns were raised regarding a number of figures, specifically:

Figure 9A: the P53 band appears to be highly similar to the p53 band of Figure 6C of an article that was simultaneously under consideration [2].

Figure 9B: the B-actin band appears to highly similar to the Actin band in Figure 4D of a previously published article [1] and to the Actin band in Figure 6C of [2].

Figure 6 appears to contain overlapping panels with Figure 8 of an article that was simultaneously under consideration [2].

The Editors-in-Chief therefore no longer have confidence in the results and conclusions reported in this article.

The authors have not responded to correspondence regarding this retraction.
